# Application of UHPLC Fingerprints Combined with Chemical Pattern Recognition Analysis in the Differentiation of Six *Rhodiola* Species

**DOI:** 10.3390/molecules26226855

**Published:** 2021-11-13

**Authors:** Didi Ma, Lijun Wang, Yibao Jin, Lifei Gu, Xiean Yu, Xueqing Xie, Guo Yin, Jue Wang, Kaishun Bi, Yi Lu, Tiejie Wang

**Affiliations:** 1 School of Pharmacy, Shenyang Pharmaceutical University, Shenyang 110016, China; madidiwy@163.com (D.M.); x2289951094@163.com (X.X.); kaishunbi.syphu@gmail.com (K.B.); 2 Shenzhen Institute for Drug Control, Shenzhen 518057, China; wanglijun8706@hotmail.com (L.W.); jinyibao2006@126.com (Y.J.); liphiegu@gmail.com (L.G.); yuxieanalj@126.com (X.Y.); ayinguoa@126.com (G.Y.); wangjuepha@126.com (J.W.); 3 NMPA Key Laboratory for Quality Research and Evaluation of Traditional Chinese Medicine, Shenzhen Institute for Drug Control, Shenzhen 518057, China; 4 Shenzhen Key Laboratory of Drug Quality Standard Research, Shenzhen Institute for Drug Control, Shenzhen 518057, China

**Keywords:** *Rhodiola*, UHPLC fingerprint, chemical pattern recognition, quality evaluation

## Abstract

*Rhodiola*, especially *Rhodiola crenulate* and *Rhodiola rosea*, is an increasingly widely used traditional medicine or dietary supplement in Asian and western countries. Because of the phytochemical diversity and difference of therapeutic efficacy among *Rhodiola* species, it is crucial to accurately identify them. In this study, a simple and efficient method of the classification of *Rhodiola crenulate*, *Rhodiola rosea*, and their confusable species (*Rhodiola serrata*, *Rhodiola yunnanensis*, *Rhodiola kirilowii* and *Rhodiola fastigiate*) was established by UHPLC fingerprints combined with chemical pattern recognition analysis. The results showed that similarity analysis and principal component analysis (PCA) could not achieve accurate classification among the six *Rhodiola* species. Linear discriminant analysis (LDA) combined with stepwise feature selection exhibited effective discrimination. Seven characteristic peaks that are responsible for accurate classification were selected, and their distinguishing ability was successfully verified by partial least-squares discriminant analysis (PLS-DA) and orthogonal partial least-squares discriminant analysis (OPLS-DA), respectively. Finally, the components of these seven characteristic peaks were identified as 1-(2-Hydroxy-2-methylbutanoate) β-D-glucopyranose, 4-O-glucosyl-p-coumaric acid, salidroside, epigallocatechin, 1,2,3,4,6-pentagalloyglucose, epigallocatechin gallate, and (+)-isolarisiresinol-4′-O-β-D-glucopyranoside or (+)-isolarisiresinol-4-O-β-D-glucopyranoside, respectively. The results obtained in our study provided useful information for authenticity identification and classification of *Rhodiola* species.

## 1. Introduction

*Rhodiola*, a genus of perennial herbaceous plants in the family Crassulaceae, comprises more than 100 species, at least 70 of which have been recorded in China [1]. Many species of *Rhodiola* have been used as traditional medicines or dietary supplements in Asia, Europe, and the United States to improve overall health. *Rhodiola crenulate* is the only official species recorded in the Chinese Pharmacopoeia, its root and rhizome are widely used in Tibetan medicine and traditional Chinese medicine for its observable effects, such as acute mountain sickness and fatigue resistance [2,3]. *Rhodiola rosea* is a commonly used species in western countries and is present in the market as a dietary supplement with antifatigue, antistress, and antidepressant properties [4,5]. *Rhodiola kirilowii* is recorded in the standardization of traditional Chinese medicine in Gansu province for hemostasis, alleviating pain, trauma, irregular menstruation, and dysentery [6]. Furthermore, *Rhodiola fastigata* is used for dissipating blood stasis, detumescence, and trauma; *Rhodiola yunnanensis* is used in detumescence, rheumatism, ostalgia, mastitis, furuncle, and open fracture [7]. In China, *Rhodiola crenulata* has been generally recognized as the appropriate raw materials of high quality for making various products, including medicines, functional foods, and cosmetics [8]. Like *Rhodiola crenulata*, the increasing demand for *Rhodiola rosea* in western countries has also caused its shortage. Therefore, other *Rhodiola* species such as *Rhodiola serrata*, *Rhodiola yunnanensis*, *Rhodiola kirilowii*, and *Rhodiola fastigiate* have been sold as *Rhodiola crenulate* or *Rhodiola rosea* in the market [8,9], while different species of *Rhodiola* possess different pharmacological activities. However, the morphologies of different species of commercial *Rhodiola* samples are too similar to distinguish visually [10]. Salidroside and tyrosol are commonly considered as chemical markers for quality control, including methods documented in Pharmacopoeia [2,11,12]. However, it has been reported that salidroside and tyrosol are the common components in various *Rhodiola* extracts [1]. Owing to the complex composition of *Rhodiola* species, it is insufficient to perform quality assessments using one or two chemical markers. There is an urgent need to establish a comprehensive quality evaluation method among the various *Rhodiola* species based on their integral components.

Fingerprint analysis has become a comprehensive method for quality evaluation of complex traditional Chinese medicines and plant extracts based on the holistic chemical profile obtained by various analytical techniques, such as gas chromatography (GC), high/ultra-high performance liquid chromatography (HPLC/UHPLC), nuclear magnetic resonance (NMR), and infra-red (IR) spectroscopy [13,14]. Among them, HPLC is the commonly used method for fingerprint analysis [15,16]. However, time-consuming, inability to withstand high pressure and large use of organic solvent are considered as its imperfections [17]. These problems have been successfully solved by UHPLC, which means introducing an environment-friendly approach to drug analysis achieved in a shorter run time with increasing resolution [17]. However, UHPLC fingerprints of traditional Chinese medicines often contain highly complex multivariate data that make their interpretation difficult. In recent years, chemical pattern recognition has attracted increasing attention in the fields of data mining, which can simplify complex data and extract hidden information from fingerprints [18]. Therefore, many chemical pattern recognition models have been used to reasonably distinguish the quality differences of the samples, such as hierarchical cluster analysis (HCA), principal component analysis (PCA), linear discriminant analysis (LDA), partial least-squares discriminant analysis (PLS-DA), and orthogonal partial least-squares discriminant analysis (OPLS-DA) [19,20].

In fact, fingerprint combined with chemical pattern recognition has been used in the analysis of *Rhodiola*. For example, ^1^H-NMR fingerprinting combined with PCA, PLS-DA, HCA, and gene expression programming was applied to distinguish *Rhodiola crenulate*, *Rhodiola kirilowii*, and *Rhodiola fastigiate* by Li et al. [21]. Li et al. [22] developed a method in the classification of four different species of *Rhodiola* (*Rhodiola crenulata*, *Rhodiola fastigiata*, *Rhodiola kirilowii*, and *Rhodiola brevipetiolata*) by fourier transform near-infrared spectroscopy combined with kernel extreme learning machine and PLS-DA analysis. However, a comprehensive and effective method for identification and classification of *Rhodiola crenulate*, *Rhodiola rosea*, and more kinds of confusable species in the market and finding out the potential chemical markers would be of great interest. 

In this study, *Rhodiola crenulata*, *Rhodiola serrata*, *Rhodiola yunnanensis*, *Rhodiola rosea*, *Rhodiola kirilowii*, and *Rhodiola fastigiata* were selected to investigate their quality variation using UHPLC fingerprints with chemical pattern recognition. Unsupervised (PCA) and supervised (LDA) pattern recognition methods were both applied to discriminate samples based on UHPLC fingerprints. The characteristic peaks responsible for the classification were obtained by LDA, and their distinguishing ability was verified by PLS-DA and OPLS-DA models, respectively. Afterward, UHPLC-Q-TOF-MS/MS was applied to identify the obtained characteristic peaks. The proposed strategy provides a more comprehensive method to evaluate the quality of various *Rhodiola* species.

## 2. Results and Discussion

### 2.1. Optimization of Sample Preparation

The extraction conditions for *Rhodiola* were optimized by comparing different extraction methods (ultrasonic extraction and refluxing extraction), extraction solvents (water; 30/70, 50/50, 70/30 EtOH/H_2_O (*v*/*v*); and EtOH), and extraction time (15, 30, 45, and 60 min). Compared to refluxing extraction, the ultrasonic method was preferred as it was more efficient, faster, easier to be operated, and required lower extraction temperature and less solvent (Figure S1). As can be seen in Figure S2, the 30/70 EtOH/H_2_O (*v*/*v*) should be selected as an extraction solvent based on the peak numbers and areas. For extraction time, there was a rapid increase in peak numbers and areas from 15 min to 45 min, but after 45 min, the extraction efficiency increased slowly (Figure S3). Results suggested that samples were optimally extracted by the ultrasonic method with 30/70 EtOH/H_2_O (*v*/*v*) for 45 min.

### 2.2. Optimization of the Chromatographic Conditions

To obtain useful chemical information and better separation, several parameters including detection wavelength (205, 225, 251, 265, 275, and 360 nm), mobile phase composition (acetonitrile/water, methanol/water, acetonitrile/0.1% aqueous formic acid) and temperature (25, 30, 35 and 40 °C) were optimized. The wavelength of 275 nm was selected as a suitable detection wavelength based on the flat baseline, more detectable peaks, and larger response values (Figure S4). As can be seen in Figure S5, the acetonitrile/water system had better resolution than methanol/water. Meanwhile, the addition of 0.1% (*v*/*v*) formic acid in water improved the resolution of target compounds. Furthermore, the increase in temperature from 25 °C to 40 °C resulted in improved peak shape and resolution (Figure S6). Therefore, acetonitrile/0.1% aqueous formic acid was considered as the optimum mobile phase and the temperature was set at 40 °C for further studies.

### 2.3. Methodology Validation

The analytical method was validated through precision, repeatability, and stability, respectively. The system precision was determined by six consecutive injections of the same sample solution. Six independent samples were prepared in parallel for the evaluation of repeatability. The stability was assessed by repeatedly analyzing one sample solution after being stored at room temperature for 0, 2, 4, 8, 12, and 24 h, respectively. For instrument precision, repeatability of the method, and stability of the sample solution, all relative standard deviations (RSDs) including retention times (RTs) and peak areas were <3% (See Supplementary Materials, Table S1), which indicated that the method is suitable for fingerprint analysis.

### 2.4. UHPLC Fingerprint Analysis and Similarity Evaluation

**Figure 1:**
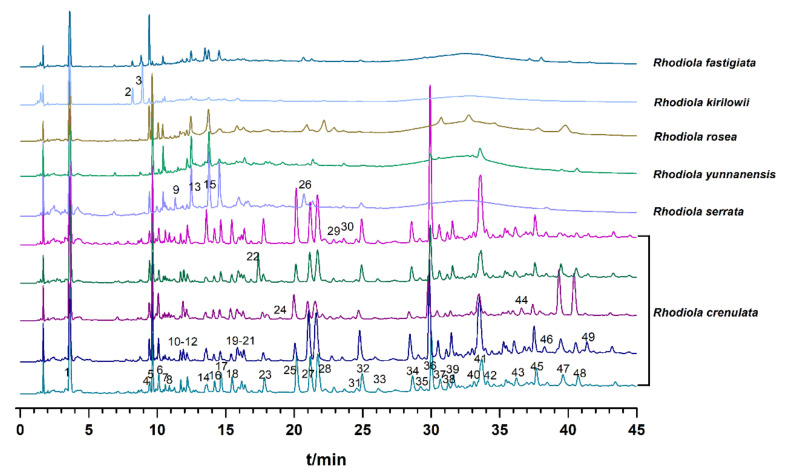
Typical UHPLC fingerprints of *Rhodiola* from six different species.

The fingerprints of 159 batches of samples were established under optimized conditions. In fingerprints, a total of 49 peaks were obtained, and the typical chromatograms of *Rhodiola crenulate*, *Rhodiola serrata*, *Rhodiola yunnanensis*, *Rhodiola rosea*, *Rhodiola kirilowii*, and *Rhodiola fastigiata* were presented in Figure 1. Based on the sample fingerprints and reference fingerprint similarities were calculated. The results of similarity values were shown in Table S2, and they were in the range of 0.785 to 0.967 for *Rhodiola crenulate*, 0.393 to 0.597 for *Rhodiola serrata*, 0.595 to 0.623 for *Rhodiola yunnanensis*, 0.449 to 0.459 for *Rhodiola rosea*, 0.516 to 0.517 for *Rhodiola kirilowii*, and 0.522 for *Rhodiola fastigiata*. *Rhodiola crenulate* could be preliminarily distinguished from others based on similarity values, while no significant difference was observed among other species. Therefore, similarity evaluation was not efficient enough for classifying different *Rhodiola* species. Consequently, the chemical pattern recognition method was employed to assess the variation in quality.

### 2.5. Chemical Pattern Recognition Analysis

#### 2.5.1. Principal Component Analysis

**Figure 2:**
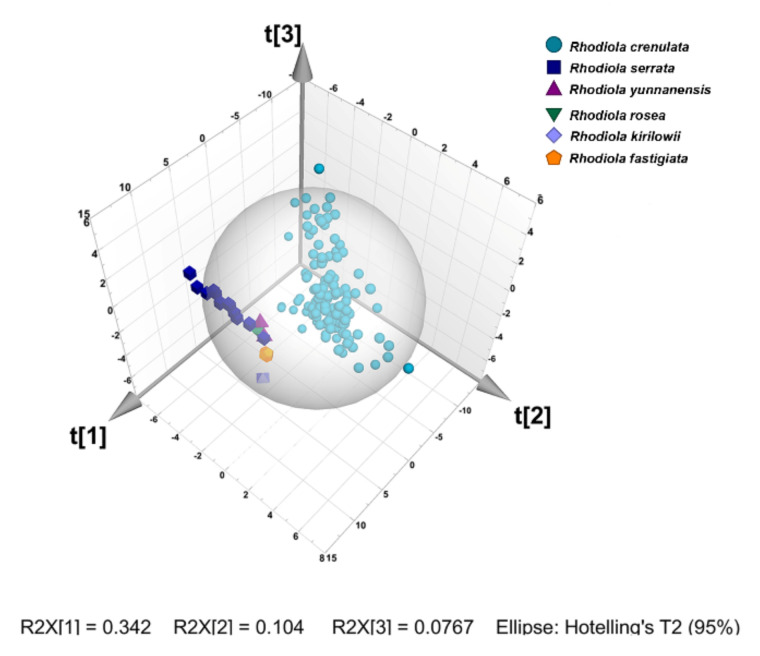
Score plot of PCA on the first three principal components for *Rhodiola* samples.

PCA is widely used for data compression and information extraction by reducing a large number of variables to a small set without losing much information [23]. In this study, PCA was performed based on the data matrix with dimensions 159 (samples) × 49 (peaks). The analysis showed that the standardized peak area matrix was transformed into principal components (PCs) comprising a new set of seven orthogonal variables. The first three PCs were extracted and explained 34.2%, 10.4%, and 7.6% of the total variation, respectively. Figure 2 showed the scores of the first three PCs, illustrating the distribution of the samples from six *Rhodiola* species. The *Rhodiola crenulate* could be distinguished clearly from others. However, appropriate visualization and differentiation could not be observed for *Rhodiola serrata*, *Rhodiola yunnanensis*, *Rhodiola rosea*, *Rhodiola kirilowii*, and *Rhodiola fastigiata*. The result revealed that PCA was not able to provide an accurate classification for the selected species of *Rhodiola*. Consequently, the supervised method was needed to find out the specific variation to classify the six species accurately.

#### 2.5.2. Linear Discriminant Analysis

LDA is a supervised pattern recognition method is frequently used for feature extraction and classification of multivariate data [20]. This procedure generates a set of discriminant functions based on linear combinations of the predictor variables that provide the best discrimination among the different groups [24]. In this work, stepwise LDA was applied to classify *Rhodiola* according to the related species. The model (function) was obtained using the training set consisting of 85 samples, while 74 samples were used as the testing set to validate the predictive ability. Seven characteristic variables were selected to generate the discriminant functions, which denoted the areas of the peaks 2, 4, 5, 7, 13, 36, and 37, respectively. The six discriminant functions were generated from six different species were as follows:

A = −0.595 × X_2_ + 3.597 × X_4_ + 2.574 × X_5_ + 0.601 × X_7_ − 0.663 × X_13_ + 0.410 × X_36_ − 1.243 × X_37_ − 15.552

B = 0.133 × X_2_ + 2.032 × X_4_ − 0.409 × X_5_ − 0.013 × X_7_ + 10.028 × X_13_ + 0.002 × X_36_ + 0.419 × X_37_ − 38.376

C = −0.268 × X_2_ − 4.616 × X_4_ + 6.611 × X_5_ + 13.020 × X_7_ + 2.488 × X_13_ + 0.113 × X_36_ − 10.005 × X_37_ − 47.297

D = −2.890 × X_2_ + 11.260 × X_4_ + 0.150 × X_5_ + 2.366 × X_7_ + 4.230 × X_13_ − 0.804 × X_36_ + 8.164 × X_37_ − 51.115

E = 107,875.876 × X_2_ + 0.641 × X_4_ + 0.061 × X_5_ − 0.399 × X_7_ + 1.092 × X_13_ + 0.018 × X_36_ − 0.029 × X_37_ − 106,799.405

**Figure 3:**
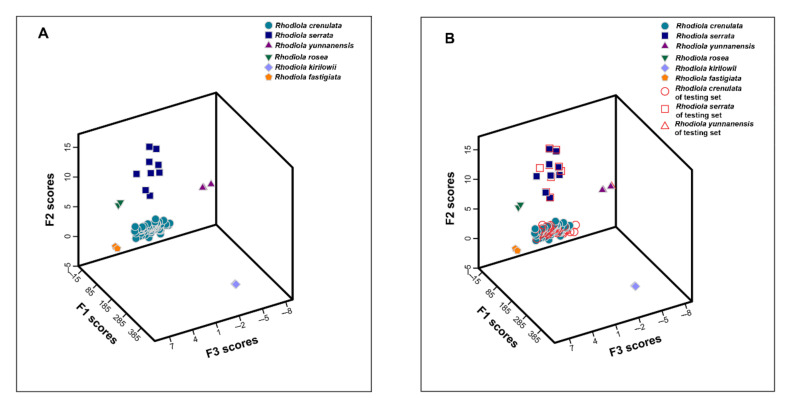
LDA score plot of training set samples (**A**); training set and testing set samples (**B**).

F = 31,321.634 × X_2_ + 21.247 × X_4_ − 2.209 × X_5_ + 1.631 × X_7_ − 1.527 × X_13_ − 0.092 × X_36_ + 0.306 × X_37_ − 9058.316

where A denotes samples from *Rhodiola crenulata*, B denotes samples from *Rhodiola serrata*, C denotes samples from *Rhodiola yunnanensis*, D denotes samples from *Rhodiola rosea*, E denotes samples from *Rhodiola kirilowii*, F denotes samples from *Rhodiola fastigiata*, and X_i_ denotes the variables. When assigning a sample, the peak area values of the seven variables can be put into the six functions, and the sample belongs to the cluster where the calculated value of the function is the highest. The classification result for the training set was shown in a scatter plot of the samples in 3D space (Figure 3A) defined by the first three discriminant functions. All the samples were excellently divided into six clusters, demonstrating a remarkable difference among the six types. The leave-one-out cross-validation method, employed as an internal tool to predict the accuracy of the model, classified 98.8% of the samples correctly. To validate the performance of the established model, 74 batches of external testing set samples were distinguished by discriminant functions. As shown in Figure 3B, the samples were accurately separated into the six related clusters that further proved the success of the established model.

#### 2.5.3. Verification of Distinguishing Ability of Characteristic Variables

**Figure 4:**
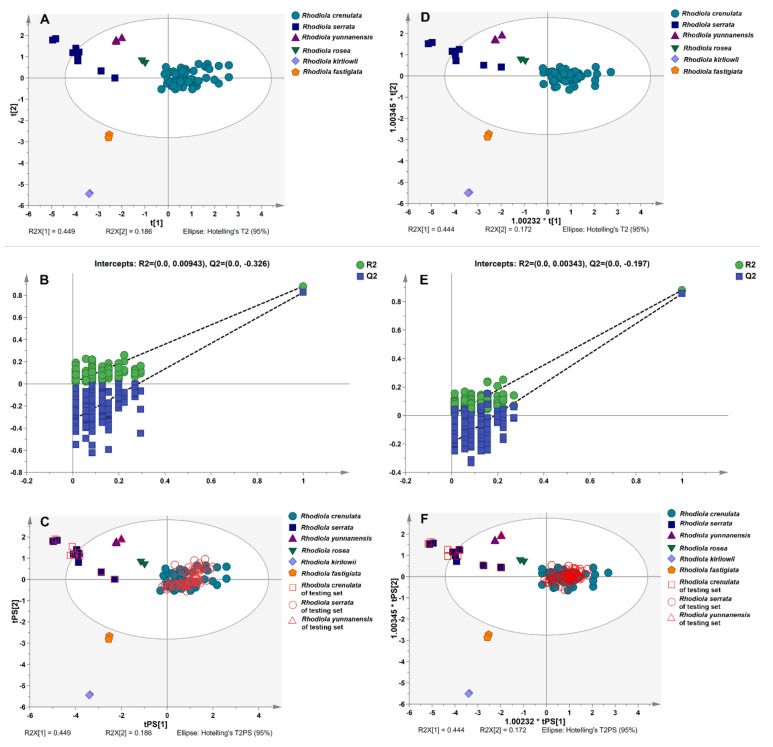
PLS-DA/OPLS-DA score plot of training set samples (**A**,**D**); permutation test result (**B**,**E**); score plot of training set and testing set samples (**C**,**F**).

For verifying whether the above-mentioned variables have the ability to discriminate different *Rhodiola* species as potential chemical markers, PLS-DA and OPLS-DA models were also generated. In PLS-DA, the data matrix of 85 (samples) × 7 (characteristic peaks) was formed to construct the classification model. The values of R^2^X, R^2^Y, and Q^2^ were 0.981, 0.736, and 0.505 at a confidence level of 95%, respectively, which showed that the established model had a good fitting and predictive ability. The score plot was shown in Figure 4A, the separation of six different *Rhodiola* species could be clearly observed. The 200 permutation tests were performed and the vertical intercept values of R^2^ and Q^2^ were 0.00943 and −0.326 (Figure 4B), respectively, indicating that the developed model avoided the problem of over-fitting and showed a good prediction. The prediction results in the testing set showed that the samples were correctly classified into their corresponding species clusters (Figure 4C).

After that, seven variables were used to construct the OPLS-DA model. At a confidence level of 95%, the values of R^2^X, R^2^Y, and Q^2^ were 0.980, 0.736, and 0.663, respectively, reflecting the established model had goodness of fit and great predictability. The score plot (Figure 4D) indicated that the OPLS-DA model could successfully provide a distinct classification of samples. The 200 permutation tests revealed that the model was not over-fitting (Figure 4E), and all the samples in the testing set were correctly classified into their related groups (Figure 4F).

Eventually, based on the seven characteristic peaks, LDA, PLS-DA, and OPLS-DA models achieved the excellent classification of samples from six *Rhodiola* species, respectively. Therefore, peaks 2, 4, 5, 7, 13, 36, and 37 were critical for the classification of *Rhodiola crenulata*, *Rhodiola serrata*, *Rhodiola yunnanensis*, *Rhodiola rosea*, *Rhodiola kirilowii*, and *Rhodiola fastigiate*, and could be selected as chemical markers for the quality evaluation of *Rhodiola* from different species.

### 2.6. Identification of the Characteristic Peaks

Seven characteristic peaks were identified from the six kinds of sample solutions by UHPLC-Q-TOF-MS/MS. The peaks were identified or tentatively assigned by comparing with the reference compounds and/or matching the empirical molecular formulae and mass fragments with those of the known compounds published in the literature. The component of peak 5 was identified as salidroside [25], with [M-H]^−^ ion at *m*/*z* 299.1134 (C_14_H_20_O_7_), and fragment ions at *m*/*z* 179.0553 [M-H-C_8_H_8_O]^−^, 119.0498 [M-H-Glu-H_2_O]^−^. The component of peak 13, with [M-H]^−^ ion at *m*/*z* 457.0773 (C_22_H_18_O_11_) and fragment ions at *m*/*z* 305.0667 [M-H-C_7_H_5_O_4_]^−^, 287.0568 [M-H-C_7_H_5_O_4_-H_2_O]^−^, 169.0133 [M-H-C_15_H_12_O_6_]^−^ and 125.0238 [M-H-C_7_H_5_O_4_-C_9_H_8_O_4_]^−^, was identified as epigallocatechin gallate [26]. Similarly, the components of peaks 2, 4, 7, 36, 37 were identified as 1-(2-Hydroxy-2-methylbutanoate) β-D-glucopyranose, 4-O-glucosyl-p-coumaric acid, epigallocatechin, 1,2,3,4,6-pentagalloyglucose, and (+)-isolarisiresinol-4′-O-β-D-glucopyranoside or (+)-isolarisiresinol-4-O-β-D-glucopyranoside, respectively [25,26,27]. The MS/MS spectrum fragment ions of them were shown in Table 1, and the chemical structures can be seen in Figure S7. Among them, salidroside (peak 5) and 1,2,3,4,6-pentagalloyglucose (peak 36) were unambiguously identified by comparison with the reference substances. 

## 3. Materials and Methods

### 3.1. Materials and Reagents

HPLC-grade ethanol (EtOH) and acetonitrile were obtained from Merck (Darmstadt, Germany). Formic acid was purchased from Aladdin Chemicals (Shanghai, China). Reference standard of salidroside was obtained from National Institutes for Food and Drug Control (Beijing, China). And 1,2,3,4,6-pentagalloyglucose was purchased from Chengdu Chroma-Biotechnology (Chengdu, China). The water was purified by a Milli-Q water purification system (Billerica, MA, USA). The roots and rhizomes of 159 batches of samples were collected from China and included 131 batches of *Rhodiola crenulata*, 18 batches of *Rhodiola serrata*, 4 batches of *Rhodiola yunnanensis*, and 2 batches of *Rhodiola rosea*, *Rhodiola kirilowii*, *Rhodiola fastigiata*, respectively. All samples were authenticated by the traditional Chinese medicine testing department (Shenzhen Institute for Drug Control, Shenzhen, Guangdong, China). The detailed sample information is listed in Table 2.

### 3.2. Apparatus and Conditions

UHPLC analysis was performed on a DIONEX Ultimate 3000 UHPLC system (Thermo Fisher Scientific, Waltham, MA, USA), which consists of an Ultimate 3000 RS pump, Ultimate 3000 RS autosampler, Ultimate 3000 RS column compartment, Ultimate 3000 RS Diode array detector, and Chromeleon software. All separations were performed on an ACQUITY UPLC HSS T3 column (2.1 × 150 mm, 1.8 μm). The mobile phase was composed of 0.1% aqueous formic acid (*v*/*v*) (A) and acetonitrile (B) with the following gradient elution: 0 min/3% B, 2 min/5% B, 6 min/12% B, 22 min/15% B, 28.5 min/19% B, 45 min/22% B. The column temperature and flow rate were set at 40 °C and 0.3 mL/min, respectively. The injection volume was 2 µL and the detection wavelength was set at 275 nm.

Identification of the characteristic peaks from the UHPLC fingerprints was performed on a UHPLC-Q-TOF-MS/MS system. Separation was carried out on a UHPLC system (Shimadzu, Kyoto, Japan) using the same column with the same mobile phases and the same gradient conditions above-mentioned. After separation, mass spectra were acquired on the AB X500R Q-TOF mass spectrometer (AB SCIEX, Framingham, MA, USA) with an ESI source. The spectrometer was operated in full-scan TOF-MS at *m*/*z* 100–1500 and information-dependent acquisition (IDA) MS/MS modes, with negative ionization mode. The optimized parameters of mass spectrometry were: Ion Source Temperature: 550 °C; Curtain Gas: 35 psi; Ion Source Gas 1 and 2: 50 psi; Ion Spray Voltage: −4500 V; Declustering Potential: −80 V (MS and MS/MS); Collision Energy: −10 V (MS), −35 V (MS/MS); Collision Energy Spread 15 V (MS/MS); Mass Range: 100–1500 *m*/*z* (MS), 50–1500 *m*/*z* (MS/MS); Accumulation Time: 0.15 s (MS), 0.05 s (MS/MS).

### 3.3. Preparation of Sample Solutions

Dry raw materials were firstly grounded into fine powder by a high-speed pulverizer and then filtered through 50 mesh sieves. An amount of 0.2 g ground powder was accurately weighed and transferred to a 50 mL conical flask with a stopper, and 10 mL 30/70 EtOH/H_2_O (*v*/*v*) was added. After ultrasonication at room temperature for 45 min, 30/70 EtOH/H_2_O (*v*/*v*) was added to compensate for the weight loss during the extraction. The extract was centrifuged at 4000 rpm for 10 min. Then the supernatant was filtered through a 0.22 μm membrane (Nylon 66; Tianjin jinteng experimental equipment Co., Ltd., Tianjin, China) and stored at 4 °C for further experiments.

### 3.4. Data Analysis

#### 3.4.1. Similarity Analysis

The raw UHPLC chromatographic data of 159 samples were exported as *.AIA format file. Similarity analysis was performed using the software “Similarity Evaluation System for Chromatographic Fingerprint of Traditional Chinese Medicine” (Version 2004 A, Chinese Pharmacopoeia Committee). The reference fingerprint was generated automatically by the median method based on the chromatographic information of *Rhodiola crenulate* samples, and the similarity values of all the samples were then calculated.

#### 3.4.2. Chemical Pattern Recognition Analysis

All chromatographic data of 159 batches of samples were collected and integrated. The data were normalized using a Z-score transformation method (SPSS 22 software, IBM Inc., Chicago, IL, USA). Afterward, chemical pattern recognition analysis was performed using PCA, LDA, PLS-DA, and OPLS-DA. PCA, PLS-DA, and OPLS-DA were carried out by SIMCA-P 14.1 software (Umetrics AB, Umea, Sweden) and LDA was undertaken through SPSS 22. Among them, PCA is an unsupervised pattern recognition tool to simplify and visualize data by extracting only the important information from the dataset [28]. LDA, PLS-DA, and OPLS-DA are widely used as supervised pattern recognition methods, where are applied to screen out the main markers that are responsible for discrimination [29]. In supervised pattern recognition methods, the samples are usually divided into a training set and a testing set [30]. The classification model is developed by the training set and validated by the testing set.

## 4. Conclusions

In this study, a simple and efficient method was developed combining UHPLC fingerprints and chemical pattern recognition to authenticity identification and classification of *Rhodiola crenulate*, *Rhodiola rosea*, and their confusable species (*Rhodiola serrata*, *Rhodiola yunnanensis*, *Rhodiola kirilowii*, and *Rhodiola fastigiate*). The results showed that the samples could not be accurately classified into the right clusters by similarity evaluation and PCA. LDA had the authenticity identification performance and seven characteristic peaks that are responsible for the accurate classification were selected. Based on the selected characteristic peaks, PLS-DA and OPLS-DA could also accomplish accurate classification process. Therefore, the components of these peaks were identified by UHPLC-Q-TOF-MS/MS, which are suitable for the quality evaluation of *Rhodiola* species. In conclusion, the established method could be employed as a powerful tool for the classification and quality assessment of *Rhodiola* species.

## Figures and Tables

**Table 1:** Identification of the characteristic peaks of *Rhodiola* by UHPLC-Q-TOF-MS/MS in negative ion mode.

	[M-H]^-^					
Peak No.	Observed Mass (Da)	Error (ppm)	Formula	MS/MS	Identification	Type
2	279.1091	0.9	C_11_H_20_O_8_	117.0566[M-H-C_6_H_10_O_5_]^−^, 101.0242[M-H-C_6_H_10_O_6_]^−^	1-(2-Hydroxy-2-methylbutanoate) β-D-glucopyranose	Acyclic acid glycoside
4	325.0926	−1	C_15_H_18_O_8_	119.0500[M-H-Glu-CO_2_]^−^, 163.0399[M-H-Glu]^−^	4-O-glucosyl-p-coumaric acid	Phenylpropanoid
5 ^a^	299.1134	−0.8	C_14_H_20_O_7_	299.1155 [M-H]^−^, 179.0553 [Glu-H]^−^, 119.0498 [M-H-Glu-H_2_O]^−^	Salidroside	The phenethyl glycosides
7	305.0665	−0.3	C_15_H_14_O_7_	221.0470[M-H-2C_2_H_2_O]^−^, 203.0331[M-H-2C_2_H_2_O-H_2_O]^−^, 179.0348[M-H-C_6_H_6_O_3_]^−^, 165.0348[M-H-C_7_H_8_O_3_]^−^, 137.0244[M-H-C_8_H_8_O_4_]^−^, 125.0247[M-H-C_9_H_8_O_4_]^−^	Epigallocatechin	Flavonoids
13	457.0773	−1.0	C_22_H_18_O_11_	305.0667[M-H-C_7_H_5_O_4_]^−^, 287.0568[M-H-C_7_H_5_O_4_-H_2_O]^−^, 169.0133[M-H-C_15_H_12_O_6_]^−^, 125.0238 [M-H-C_7_H_5_O_4_-C_9_H_8_O_4_]^−^	Epigallocatechin gallate	Flavonoids
36 ^a^	939.1112	0.3	C_41_H_32_O_26_	939.1085[M-H]^−^, 769.0884[M-H-C_7_H_6_O_5_]^−^, 617.0785[M-H-C_7_H_6_O_5_-C_7_H_4_O_4_]^−^, 447.0578[M-H-2C_7_H_6_O_5_-C_7_H_4_O_4_]^−^, 169.0146[Galloy]^−^	1,2,3,4,6-Pentagalloyglucose	Gallic acid derivative
37	521.2028	−0.1	C_26_H_34_O_11_	491.1942[M-HCHO-H]^−^, 503.1883[M-H_2_O-H]^−^, 341.1383[M-Glu-H_2_O]^−^	(+)-isolarisiresinol-4′-O-β-D-glucopy ranoside or (+)-isolarisiresinol-4-O-β-D-glucopyranoside)	Phenylpropanoid

^a^ Identification by reference substances.

**Table 2:** The information of *Rhodiola* samples.

Sample No.	Species	Origin	Specifications
1–47/68–115	*Rhodiola crenulata*	Tibet	Processed drugs
48–53/116–122	*Rhodiola crenulata*	Sichuan	Processed drugs
54–57/123/124	*Rhodiola crenulata*	Xinjiang	Processed drugs
58–61/125/126	*Rhodiola crenulata*	Jilin	Processed drugs
62–65/127/128	*Rhodiola crenulata*	Qinghai	Processed drugs
66	*Rhodiola crenulata*	Inner Mongolia	Processed drugs
67	*Rhodiola crenulata*	Gansu	Processed drugs
129	*Rhodiola crenulata*	Yunnan	Processed drugs
130	*Rhodiola crenulata*	Guangxi	Processed drugs
131	*Rhodiola crenulata*	Liaoning	Processed drugs
132/141	*Rhodiola serrata*	Tibet	Processed drugs
133/142	*Rhodiola serrata*	Hunan	Processed drugs
134/143	*Rhodiola serrata*	Sichuan	Crude drugs
135–140/144–149	*Rhodiola serrata*	/	Crude drugs
150–153	*Rhodiola yunnanensis*	Tibet	Processed drugs
154/155	*Rhodiola rosea*	/	Crude drugs
156/157	*Rhodiola kirilowii*	/	Crude drugs
158/159	*Rhodiola fastigiata*	Tibet	Processed drugs

## Data Availability

The data are available within this article and its Supplementary Materials.

## Supplementary Materials

The following are available online, Table S1: Repeatability, precision, and stability of fingerprints in *Rhodiola* samples expressed by the relative standard deviation (RSD) of retention time (RT) and peak area, Table S2: Similarities of 159 batches of *Rhodiola* samples, Figure S1: UHPLC chromatograms of the extracts of *Rhodiola* sample with total peak numbers and total areas under different extraction methods, Figure S2: UHPLC chromatograms of the extracts of *Rhodiola* sample with total peak numbers and total areas under different extraction solvents, Figure S3: UHPLC chromatograms of the extracts of *Rhodiola* sample with total peak numbers and total areas by different extraction time, Figure S4: UHPLC chromatograms of the extract of *Rhodiola* sample in different wavelength, Figure S5: Effect of the different mobile phase composition on the separation of extract of *Rhodiola* sample, Figure S6: Effect of temperature on resolution, Figure S7: Chemical structures of seven characteristic peaks for discriminating six different *Rhodiola* species.

## Sample Availability

Samples of the *Rhodiola* are available from the authors.
