# Supplementary material for: Application of UHPLC Fingerprints Combined with Chemical Pattern Recognition Analysis in the Differentiation of Six Rhodiola Species
Source: Molecules. 2021 Nov 13;26(22):6855. doi: 10.3390/molecules26226855 (PMC8618991; doi:10.3390/molecules26226855)
Supplement: Supplementary file 1 [file molecules-26-06855-s001.zip › molecules-1454260-supplementary.pdf]

Supplementary Materials

Table S1. Repeatability, precision, and stability of fingerprints in *Rhodiola* samples expressed by the relative standard deviation (RSD) of retention time (RT) and peak area.

Peak No.	Precision		Repeatability		Stability	
	RT RSD%	Area RSD%	RT RSD%	Area RSD%	RT RSD%	Area RSD%
1	0.17	0.22	0.53	0.14	0.20	0.28
2	0.07	0.47	0.31	1.12	0.06	0.45
3	0.08	0.25	0.30	0.40	0.06	0.29
4	0.06	0.61	0.27	0.73	0.04	0.61
5	0.08	0.61	0.22	0.82	0.06	0.78
6	0.04	0.75	0.25	2.47	0.04	2.80
7	0.03	0.88	0.25	2.42	0.05	2.35
8	0.05	0.69	0.30	0.62	0.04	0.92
9	0.06	0.38	0.32	0.52	0.05	0.59
10	0.06	0.69	0.32	1.14	0.05	0.71
11	0.05	0.42	0.33	0.36	0.06	0.88
12	0.04	0.75	0.32	1.67	0.06	2.17
13	0.07	0.44	0.29	1.15	0.05	0.94
14	0.05	0.25	0.29	0.43	0.06	0.47
15	0.05	0.27	0.26	0.79	0.06	0.92
16	0.03	0.31	0.24	1.32	0.05	0.95
17	0.03	0.32	0.23	0.57	0.04	1.29
18	0.01	0.26	0.17	0.43	0.02	0.76
19	0.01	0.23	0.15	0.40	0.03	0.69
20	0.02	0.34	0.14	0.50	0.03	1.12
21	0.02	0.67	0.12	0.38	0.03	1.79
22	0.02	0.33	0.10	0.40	0.03	1.29
23	0.02	0.95	0.10	0.60	0.02	1.25
24	0.02	0.33	0.10	0.79	0.02	0.85
25	0.02	0.43	0.11	0.91	0.02	1.60
26	0.02	0.42	0.10	0.73	0.02	1.47

Table S2. Similarities of 159 batches of *Rhodiola* samples.

No.	Similarity	No.	Similarity	No.	Similarity	No.	Similarity
S1	0.914	S41	0.915	S81	0.908	S121	0.854
S2	0.948	S42	0.944	S82	0.940	S122	0.906
S3	0.873	S43	0.940	S83	0.953	S123	0.935
S4	0.945	S44	0.914	S84	0.909	S124	0.785
S5	0.949	S45	0.834	S85	0.925	S125	0.932
S6	0.955	S46	0.850	S86	0.951	S126	0.920
S7	0.902	S47	0.959	S87	0.856	S127	0.879
S8	0.930	S48	0.958	S88	0.897	S128	0.878
S9	0.919	S49	0.947	S89	0.961	S129	0.962
S10	0.912	S50	0.953	S90	0.955	S130	0.955
S11	0.963	S51	0.933	S91	0.862	S131	0.855
S12	0.859	S52	0.959	S92	0.934	S132	0.540
S13	0.928	S53	0.924	S93	0.864	S133	0.553
S14	0.826	S54	0.942	S94	0.928	S134	0.499
S15	0.934	S55	0.942	S95	0.932	S135	0.579
S16	0.928	S56	0.887	S96	0.956	S136	0.455
S17	0.862	S57	0.914	S97	0.857	S137	0.393
S18	0.880	S58	0.919	S98	0.918	S138	0.450
S19	0.896	S59	0.858	S99	0.902	S139	0.564
S20	0.932	S60	0.967	S100	0.897	S140	0.428
S21	0.950	S61	0.947	S101	0.949	S141	0.521
S22	0.930	S62	0.888	S102	0.965	S142	0.553
S23	0.938	S63	0.883	S103	0.939	S143	0.489
S24	0.940	S64	0.945	S104	0.835	S144	0.577
S25	0.929	S65	0.930	S105	0.923	S145	0.456
S26	0.921	S66	0.951	S106	0.946	S146	0.394
S27	0.914	S67	0.945	S107	0.919	S147	0.454
S28	0.953	S68	0.940	S108	0.871	S148	0.564
S29	0.933	S69	0.947	S109	0.950	S149	0.427
S30	0.905	S70	0.937	S110	0.936	S150	0.623
S31	0.946	S71	0.921	S111	0.923	S151	0.595
S32	0.904	S72	0.914	S112	0.913	S152	0.616
S33	0.893	S73	0.942	S113	0.930	S153	0.617
S34	0.922	S74	0.891	S114	0.932	S154	0.459
S35	0.808	S75	0.840	S115	0.848	S155	0.449
S36	0.943	S76	0.921	S116	0.952	S156	0.516
S37	0.861	S77	0.934	S117	0.948	S157	0.517
S38	0.953	S78	0.807	S118	0.889	S158	0.522
S39	0.940	S79	0.950	S119	0.907	S159	0.522
S40	0.922	S80	0.939	S120	0.952		

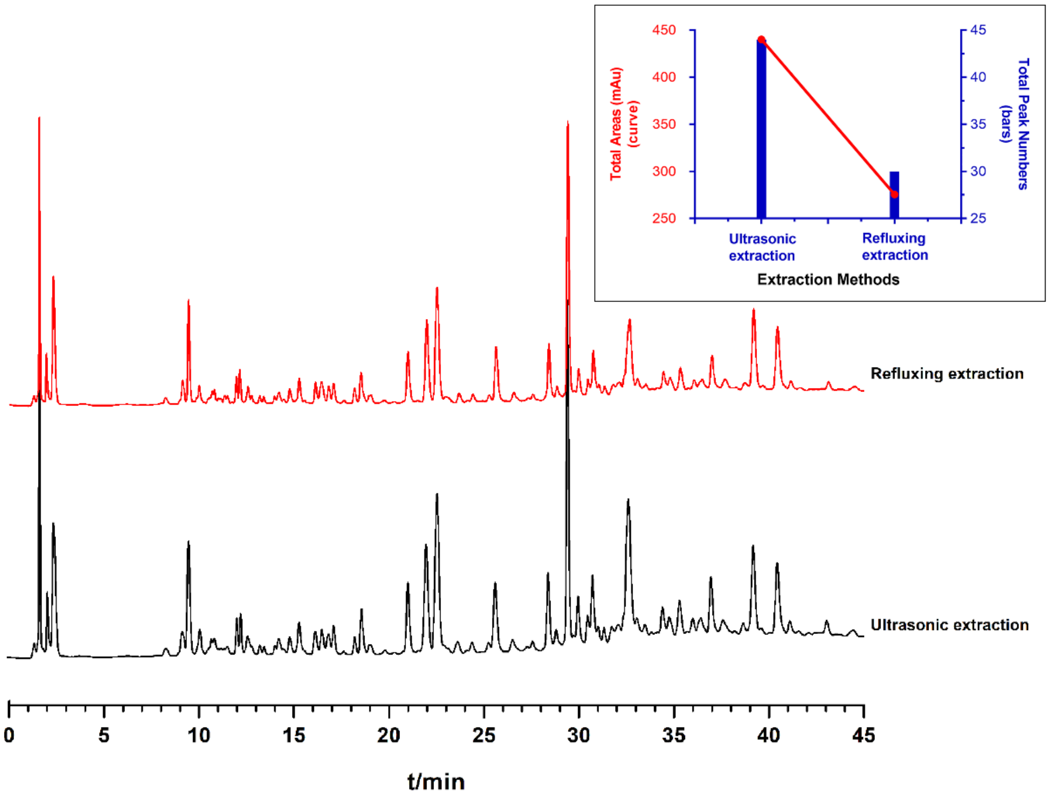

Figure S1. UHPLC chromatograms of the extracts of *Rhodiola* sample with total peak numbers and total areas under different extraction methods.

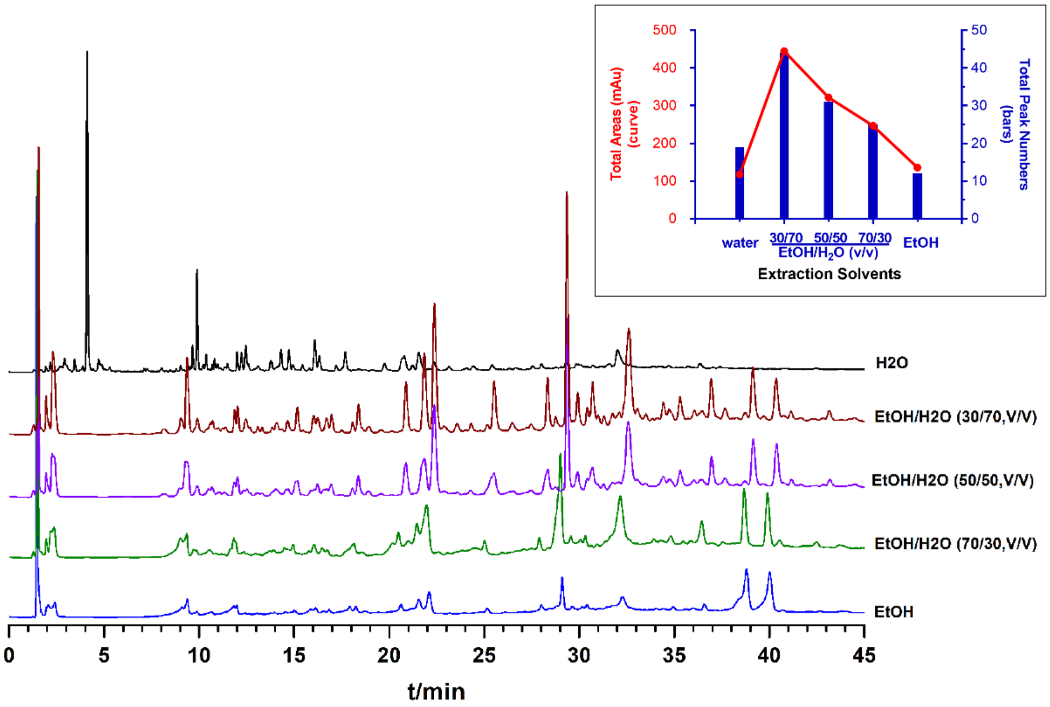

Figure S2. UHPLC chromatograms of the extracts of *Rhodiola* sample with total peak numbers and total areas under different extraction solvents.

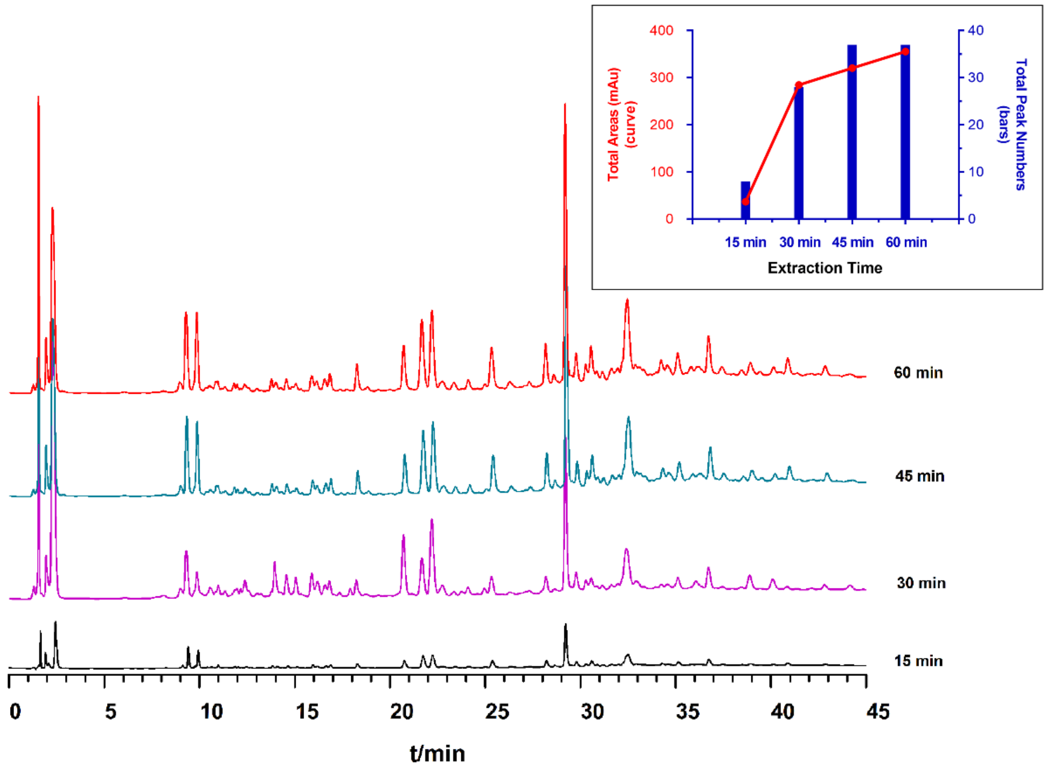

Figure S3. UHPLC chromatograms of the extracts of *Rhodiola* sample with total peak numbers and total areas by different extraction time.

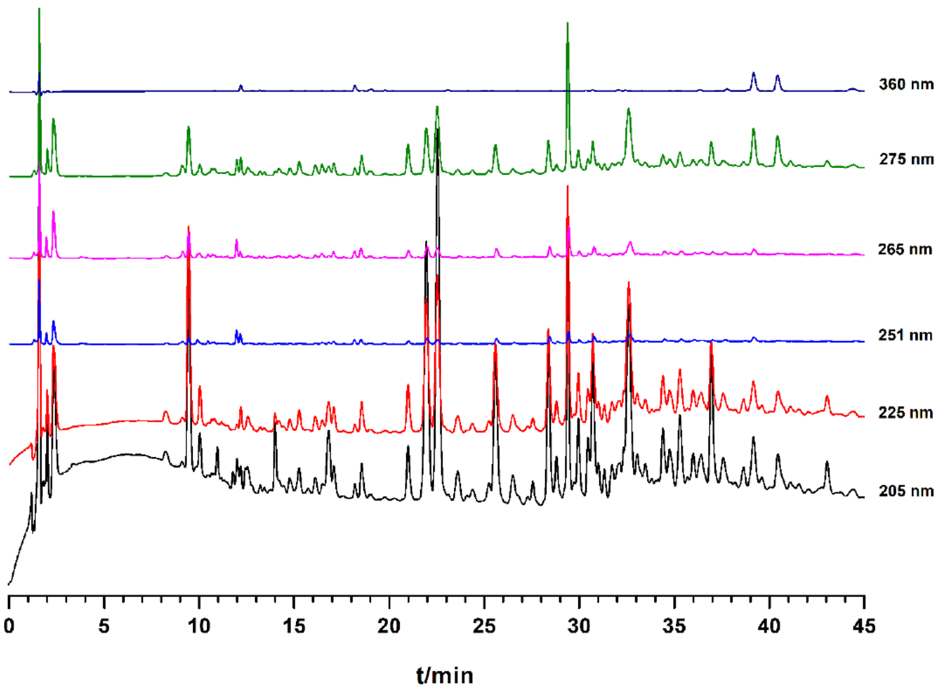

Figure S4. **U**HPLC chromatograms of the extract of *Rhodiola* sample in different wavelength.

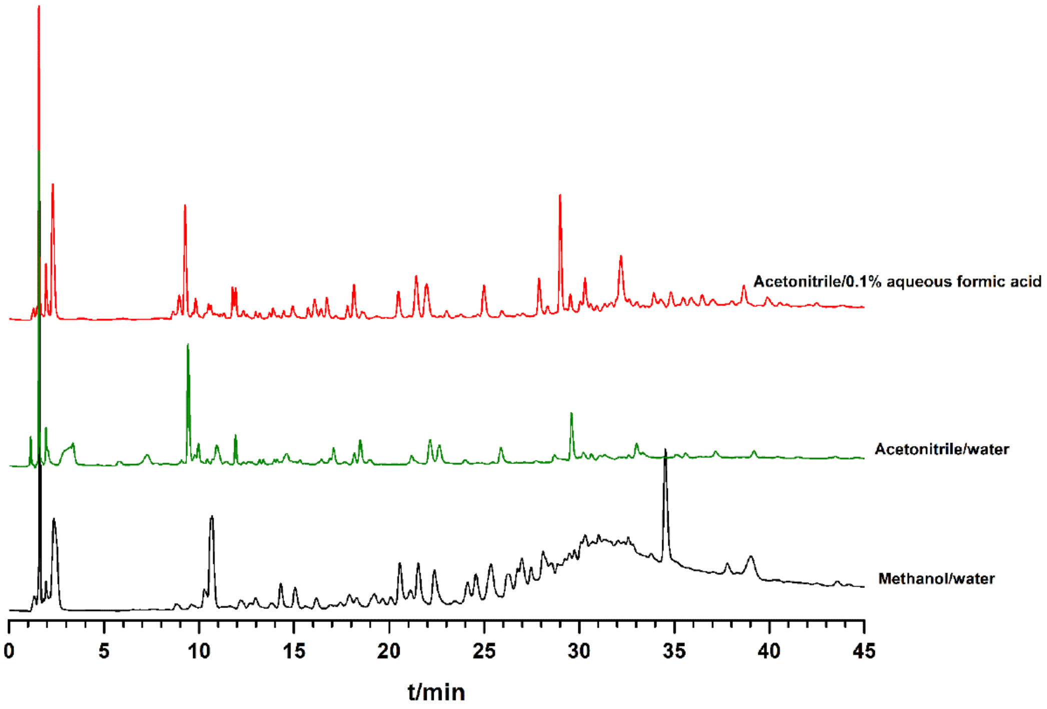

Figure S5. Effect of the different mobile phase composition on the separation of extract of *Rhodiola* sample.

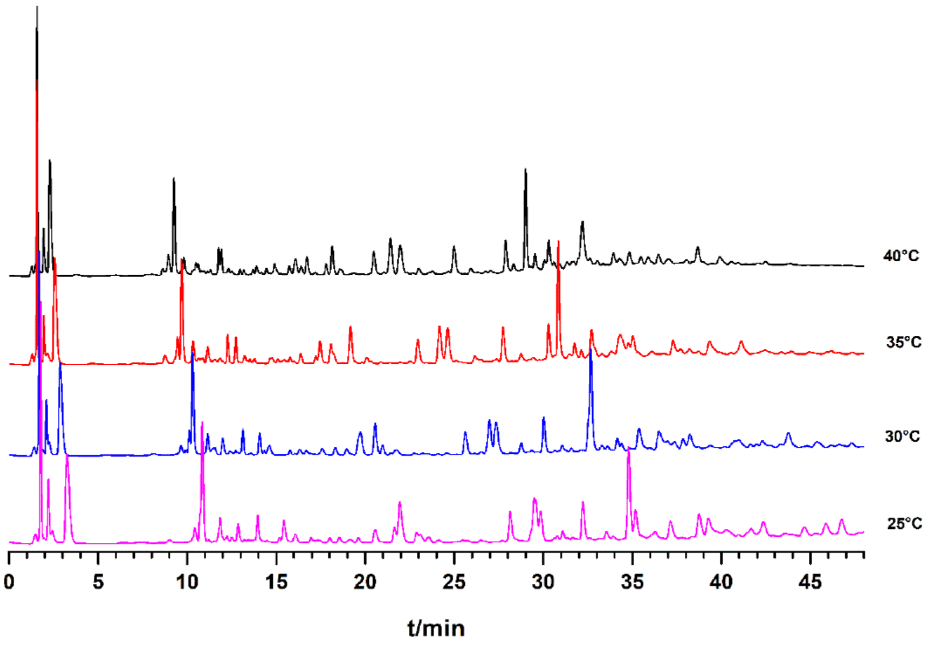

Figure S6. Effect of temperature on resolution.

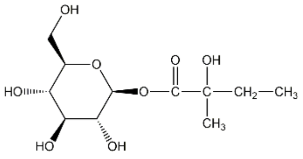

P2

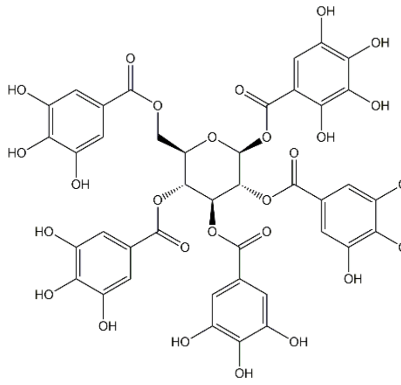

P36

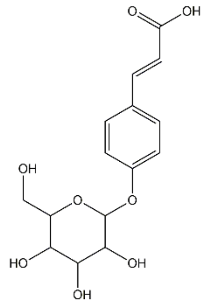

P4

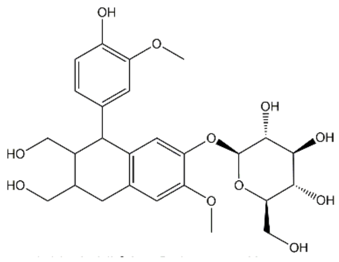

P37-1

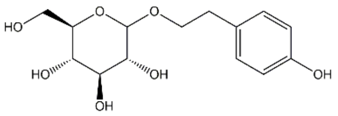

P5

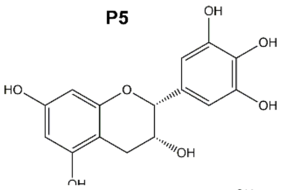

P7

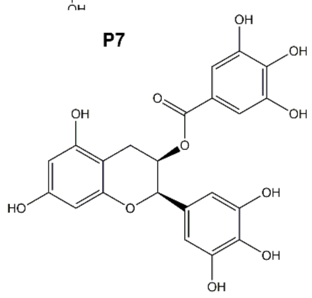

P13

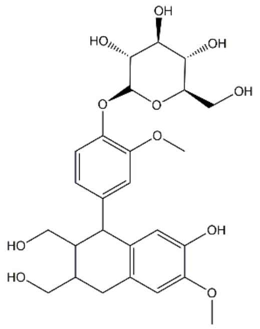

P37-2

Figure S7. Chemical structures of seven characteristic peaks for discriminating six different *Rhodiola* species.

(P5: salidroside, P36: 1,2,3,3,4,6-pentagalloy glucose, P2: 1-(2-Hydroxy-2-methylbutanoate) β-D-glucopyranose, P4:4-O-glucosyl-p-coumaric acid, P7: epigallocatechin, P13: Epigallocatechin gallate, P37-1: (+)-isolarisiresinol-4′-O-β-D-glucopyranoside; P37-2: (+)-isolarisiresinol-4-O-β-D-glucopyranoside).
